# The Role of the KLF Family in T-Cell-Mediated Regulation of Cardiovascular Diseases: Molecular Mechanisms and Therapeutic Prospects

**DOI:** 10.3390/cells15171519

**Published:** 2026-08-24

**Authors:** Shijia Wang, Xiangbin Zhu, Na Li, Kunfu Ouyang, Zhiyong Liao

**Affiliations:** 1 Department of Cardiology, Fuwai Shenzhen Hospital, Chinese Academy of Medical Sciences, Shenzhen 518057, China; wang_shijia@outlook.com; 2 Shenzhen Key Laboratory of Cardiovascular Disease, Fuwai Shenzhen Hospital, Chinese Academy of Medical Sciences, Shenzhen 518057, China; 3 Department of Cardiovascular Surgery, Peking University Shenzhen Hospital, Shenzhen 518036, China; izhuxb@163.com (X.Z.); linanana99@163.com (N.L.)

**Keywords:** Krüppel-like factor, KLF2, KLF10, T cells, regulatory T cells, cardiovascular disease, atherosclerosis, myocardial infarction, heart failure

## Abstract

**Highlights:**

**What are the main findings?**

- KLF family members regulate distinct T-cell states, including KLF2-dependent quiescence and trafficking, KLF10-dependent regulatory T-cell suppressive function and metabolic fitness, KLF4-associated effector differentiation, and KLF13-mediated inflammatory output.
- The strongest direct cardiovascular evidence currently supports a role for KLF10 within the CD4^+^ T-cell lineage in experimental atherosclerosis, with complementary functional evidence implicating Treg–macrophage interactions, whereas KLF-dependent T-cell mechanisms in myocardial infarction, myocarditis, hypertension, and heart failure remain less well validated.

**What are the implications of the main findings?**

- The KLF–T-cell axis provides a useful framework for linking transcriptional regulation of T-cell subsets to cardiovascular inflammation, tissue repair, and chronic remodeling.
- Clinical translation of KLF-dependent T-cell programs will require cell-selective and disease-stage-specific approaches with careful evaluation of off-target immune effects.

**Abstract:**

Cardiovascular diseases are increasingly recognized as immune-inflammatory disorders in which adaptive immunity shapes tissue injury, repair, and long-term remodeling. T cells are central to these processes because they integrate antigen recognition, lineage-defining transcriptional programs, tissue trafficking, cytokine production, and immunological memory. In this Review, we synthesize current evidence on the Krüppel-like factor (KLF) family as a transcriptional framework linking T-cell biology to cardiovascular disease. KLF2 primarily regulates T-cell quiescence and trafficking, KLF10 supports regulatory T-cell suppressive function and immune-metabolic fitness, KLF4 contributes to inflammatory effector differentiation, and KLF13 regulates delayed inflammatory chemokine expression and, in thymocyte models, exerts a survival-restraining effect through apoptosis-related pathways. Across atherosclerosis, myocardial infarction, myocarditis, hypertension, and heart failure, these KLF-dependent programs may influence the balance between pathogenic effector responses and protective regulatory mechanisms. The strongest direct disease-specific evidence currently supports a role for KLF10 within the CD4^+^ T-cell lineage in experimental atherosclerosis, with complementary functional evidence implicating Treg–macrophage interactions, whereas the roles of KLF-dependent T-cell programs in other cardiovascular settings remain mechanistically compelling but less fully validated. Future progress will require disease-specific T-cell-restricted models, spatially resolved immune analyses, and cell-selective translational strategies to define the therapeutic relevance of the KLF–T-cell axis.

## 1. Introduction

Cardiovascular diseases (CVDs) remain the leading cause of mortality worldwide and represent a major global health burden [1]. Although traditional risk factors such as dyslipidemia, thrombosis, and hemodynamic stress are central to disease development, accumulating evidence indicates that inflammation is a fundamental driver of disease initiation, progression, and tissue remodeling [2]. This paradigm shift has redefined CVDs as immune-inflammatory disorders rather than purely metabolic or mechanical conditions [3].

Among immune populations, T cells play a central role in coordinating adaptive immune responses through antigen recognition, lineage specification, cytokine production, and immunological memory [4]. In atherosclerosis, T cells modulate plaque development and stability [5,6]. Following myocardial infarction (MI), T cell subsets dynamically regulate inflammatory resolution and tissue repair [7,8]. In myocarditis, autoreactive T cells are key drivers of myocardial injury [9,10]. In hypertension and heart failure, chronic T cell activation contributes to vascular dysfunction and adverse cardiac remodeling [11,12]. Notably, regulatory T cells (Tregs) exert protective effects across multiple cardiovascular contexts by limiting excessive inflammation and promoting tissue repair [13,14].

The Krüppel-like factor (KLF) family consists of zinc-finger transcription factors that regulate diverse biological processes, including cell differentiation, proliferation, metabolism, and immune responses [15]. In the immune system, KLFs orchestrate leukocyte development, activation thresholds, trafficking behavior, and lineage commitment [15]. Several KLF members have emerged as critical regulators of T-cell biology: KLF2 governs T cell quiescence and trafficking [16], KLF10 regulates TGF-β signaling and Treg function [17,18,19], KLF4 promotes inflammatory differentiation programs [20], and KLF13 regulates delayed chemokine expression and, in thymocyte models, exerts a survival-restraining effect through apoptosis-related pathways [21].

Despite increasing recognition of KLFs in cardiovascular biology, most studies have focused on non-immune cells such as endothelial cells and macrophages. The role of KLF-regulated T-cell programs in cardiovascular disease remains incompletely defined. Because the strength of evidence varies substantially across KLF family members and cardiovascular disease contexts, we distinguish among three levels of evidence throughout this Review. Direct disease-specific evidence refers to cardiovascular studies in which manipulation of a KLF within a defined T-cell lineage is causally linked to a disease phenotype. Importantly, this designation reflects the lineage specificity of the experimental model and does not imply causality within a narrower T-cell subset unless that subset was specifically targeted. Emerging mechanistic evidence refers to well-established KLF-dependent T-cell functions that are biologically relevant to cardiovascular disease but have not yet been directly validated in disease-specific T-cell-restricted models. Hypothesis-generating evidence refers to relationships inferred from general T-cell biology, non-T-cell studies, or broader cardiovascular immune phenotypes. This distinction is intended to prevent mechanistic plausibility from being interpreted as established disease causality.

Given that T cell function is highly dependent on transcriptional regulation of activation, migration, and suppressive capacity, understanding how KLFs shape T cell responses may provide critical insights into immune-mediated cardiovascular pathology. In this Review, we synthesize current knowledge of the KLF–T-cell axis and its role in cardiovascular diseases, with an emphasis on molecular mechanisms and therapeutic implications.

**Literature search and evidence classification.** Relevant literature was identified through targeted PubMed searches and reference-list screening, updated through 19 August 2026. As this is a narrative review, evidence was categorized as direct disease-specific, emerging mechanistic, or hypothesis-generating according to the degree of T-cell-lineage- and cardiovascular disease-specific causal validation.

## 2. The KLF Family: Biological Overview and Scope of This Review

Krüppel-like factors (KLFs) are a family of transcription factors characterized by three conserved C2H2 zinc-finger domains at the C-terminus, which mediate binding to GC-rich DNA sequences [15,22,23]. The N-terminal regions are highly variable and enable interaction with diverse co-activators and co-repressors, thereby conferring context-dependent transcriptional regulation [22,23]. This modular structure allows KLFs to regulate overlapping gene networks while exerting distinct biological effects depending on cellular context.

KLF family members are involved in a broad spectrum of physiological processes, including epithelial differentiation, hematopoiesis, metabolism, and vascular homeostasis [15]. In the cardiovascular system, KLFs have been extensively studied in endothelial and smooth muscle cell biology, where they regulate vascular tone, inflammation, and remodeling [24,25,26,27,28,29,30]. However, their roles in immune regulation are increasingly recognized as equally important.

Evidence supporting T-cell-intrinsic functions differs substantially among KLF family members. This Review primarily focuses on KLF2, KLF4, KLF10, and KLF13 because these factors have comparatively well-defined mechanistic roles in T-cell biology and provide complementary examples of KLF-dependent regulation of lymphocyte homeostasis, regulatory function, inflammatory differentiation, and immune effector programs. Their molecular and functional roles are discussed in detail in Section 3.

Other KLF family members, including KLF5, KLF6, KLF14, and KLF15, have been implicated in cardiovascular homeostasis, metabolism, or inflammatory regulation, but their T-cell-specific functions in cardiovascular disease remain less clearly defined. They are therefore considered primarily as emerging candidates and knowledge gaps rather than as equally established components of the KLF–T-cell axis. Disease- and T-cell-restricted studies will be required to determine whether these factors directly regulate cardiovascular immune responses.

## 3. T-Cell-Intrinsic Functions of Prioritized KLF Family Members

### 3.1. KLF2: Maintenance of Quiescence and Lymphocyte Trafficking

KLF2 is the most extensively characterized KLF family member in T-cell biology and serves as a central regulator of T cell quiescence. Enforced KLF2 expression suppresses c-Myc activity and limits spontaneous T-cell activation, supporting a central role for KLF2 in maintaining mature T-cell quiescence [31]. These findings helped establish quiescence as an actively regulated transcriptional program rather than a passive default state.

Beyond quiescence, KLF2 has a well-defined role in lymphocyte trafficking. It regulates the expression of key homing receptors, including CD62L and sphingosine-1-phosphate receptor 1 (S1P1), thereby controlling lymphocyte egress and recirculation [16,32]. KLF2 also suppresses inappropriate expression of inflammatory chemokine receptors, ensuring correct tissue localization of naïve T cells [33]. Accordingly, KLF2 deficiency causes marked abnormalities in lymphocyte localization and recirculation, while exerting comparatively limited effects on proliferative competence [34].

KLF2 also contributes to immune tolerance through effects on regulatory and helper T-cell differentiation. It facilitates the generation of inducible Tregs and regulates the migratory positioning of Tregs in peripheral tissues, thereby influencing where their suppressive activity is exerted [35,36]. In addition, KLF2 restricts T follicular helper cell differentiation, further supporting its broader role in maintaining T-cell homeostasis and limiting inappropriate immune activation [37].

Importantly, most of these mechanistic findings were established in general lymphocyte, developmental, or non-cardiovascular immune models and should not be interpreted as direct evidence that KLF2 controls T-cell behavior in cardiovascular disease.

### 3.2. KLF10: TGF-β Responsiveness, Regulatory Function, and Metabolic Adaptation

KLF10 has emerged as an important context-dependent regulator of T cell tolerance and function. In the CD4^+^ T cell/Treg compartment, KLF10 is required for optimal Treg suppressive activity, as KLF10-deficient Tregs exhibit impaired suppressor function despite preserved Foxp3 expression, together with altered expression of immune-regulatory mediators such as TGF-β1 [17]. These findings indicate that preservation of Foxp3 expression alone does not ensure full Treg functional competence.

KLF10 also regulates TGF-β responsiveness in a cell-subset-specific manner. In CD8^+^ T cells, KLF10 controls the expression of transforming growth factor-β receptor II and thereby modulates downstream TGF-β signaling [18]. This observation is important because it indicates that KLF10-dependent regulation of TGF-β signaling should not be attributed uniformly to Tregs but instead varies according to T-cell lineage and activation context.

More recent evidence has linked KLF10 to T-cell immunometabolism. Klf10 deletion within the CD4^+^ T-cell lineage is associated with impaired Treg metabolic fitness and mobilization under inflammatory metabolic stress [19]. KLF10 may therefore connect transcriptional regulation of immune tolerance with the metabolic adaptation required to maintain Treg function in unfavorable inflammatory environments. The relevant metabolic pathways and their integration with TGF-β signaling are discussed further in Section 6.

### 3.3. KLF4: Inflammatory Differentiation and Proliferative Regulation

KLF4 contributes primarily to inflammatory effector differentiation and activation-associated regulation in T cells. It promotes the differentiation of IL-17-producing CD4^+^ T cells through a mechanism reported to be at least partly independent of the canonical lineage-defining factor RORγt [20]. This finding identifies KLF4 as an additional transcriptional regulator of the Th17-associated program rather than merely a downstream component of the conventional RORγt pathway.

KLF4 has also been implicated in thymocyte proliferative regulation, although this effect appears to depend on developmental and cellular context [38]. Collectively, the available evidence suggests that KLF4 contributes to inflammatory differentiation and context-dependent proliferative regulation. However, compared with KLF2 and KLF10, the downstream transcriptional network controlled by KLF4 in distinct T-cell subsets remains less completely defined.

### 3.4. KLF13: Delayed Chemokine Induction and Survival Restraint

KLF13 regulates at least two distinct aspects of T-cell biology that should be considered separately. First, KLF13, originally described as RFLAT-1, promotes the delayed transcriptional induction of RANTES/CCL5 following T-cell activation [39]. This function links KLF13 to the later phase of inflammatory chemokine production rather than to the initial activation response.

Second, KLF13 influences apoptosis and lymphocyte survival. Importantly, the available genetic evidence does not support a survival-promoting role. Klf13-deficient thymocytes exhibit reduced spontaneous and activation-induced apoptosis, prolonged survival, and increased expression of the anti-apoptotic protein BCL-XL [21]. These findings indicate that KLF13 normally exerts a survival-restraining or pro-apoptotic effect in this developmental context. The chemokine-regulatory function of KLF13 should therefore be distinguished from its effect on apoptosis, and neither function should be interpreted as direct evidence that KLF13 promotes the persistence of mature effector T cells in cardiovascular tissues.

Taken together, these findings demonstrate functional specialization among T-cell-relevant KLF family members. KLF2 primarily maintains quiescence and appropriate lymphocyte trafficking and tissue distribution, KLF10 supports regulatory function, TGF-β responsiveness, and metabolic fitness, KLF4 contributes to inflammatory differentiation and proliferative regulation, and KLF13 coordinates delayed chemokine expression with survival-restraining apoptotic control. These T-cell-intrinsic functions provide the mechanistic foundation for evaluating their potential relevance across different cardiovascular disease contexts in subsequent sections.

**Figure 1:**
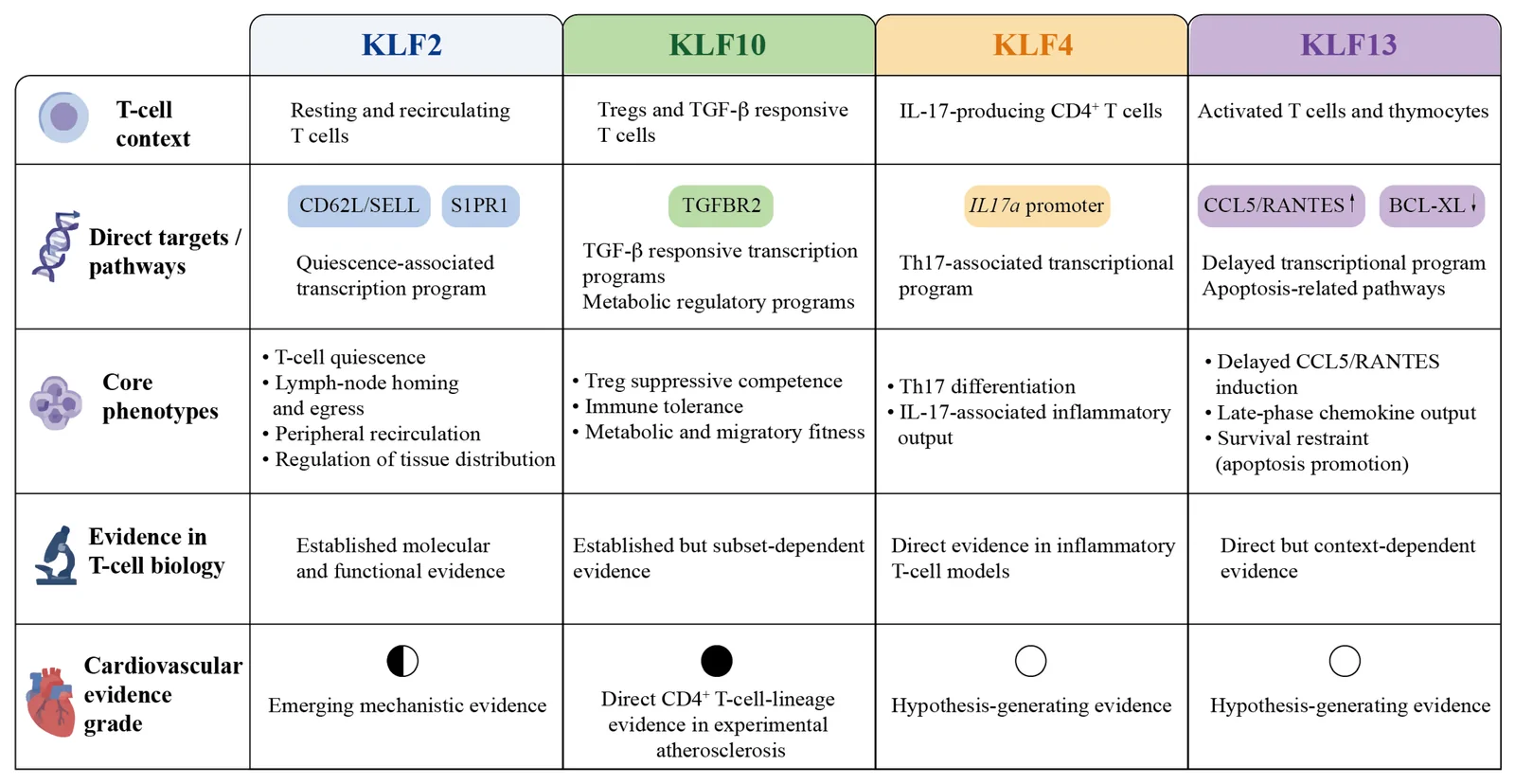
Functional spectrum and evidence hierarchy of prioritized KLF family members in T-cell biology. KLF2 regulates quiescence-associated transcription and SELL/CD62L- and S1PR1-dependent trafficking and recirculation. KLF10 supports TGF-β-responsive transcription, Treg suppressive competence, and metabolic and migratory fitness, with TGFBR2 regulation demonstrated in a subset-dependent context. KLF4 directly promotes *Il17a* transcription and contributes to Th17-associated inflammatory differentiation. KLF13 regulates delayed CCL5/RANTES expression and represses the anti-apoptotic protein BCL-XL, thereby exerting a survival-restraining effect; the latter evidence is derived predominantly from thymocyte models. Evidence within general T-cell biology ranges from established molecular or functional evidence to context-dependent observations, whereas cardiovascular evidence is strongest for KLF10 manipulation within the CD4^+^ T-cell lineage in experimental atherosclerosis; Treg-focused functional studies support involvement of regulatory T-cell mechanisms without establishing exclusively Treg-intrinsic causality. Filled, half-filled, and open circles denote direct disease-specific evidence, emerging mechanistic evidence, and hypothesis-generating cardiovascular evidence, respectively. Cardiovascular evidence grades refer specifically to KLF-dependent T-cell mechanisms rather than to the broader cardiovascular functions of individual KLF family members. Upward and downward arrows indicate increased and decreased expression, respectively; colors are used only to distinguish individual KLF modules and do not encode evidence strength.

Figure 1 and Table 1 summarize the T-cell contexts, molecular programs, and cardiovascular evidence levels of the prioritized KLF family members.

## 4. T-Cell-Mediated Immune Mechanisms in Cardiovascular Diseases

T-cell responses in cardiovascular disease are strongly shaped by disease context, tissue localization, and stage of injury. Rather than exerting uniformly protective or pathogenic effects, individual T-cell subsets may adopt distinct functions depending on the local inflammatory environment and the timing of immune activation. Atherosclerosis is characterized predominantly by chronic plaque-associated immune activation, whereas myocardial infarction involves a temporally ordered transition from inflammation to repair. Myocarditis is driven more directly by antigen-specific autoreactive responses, while hypertension and heart failure are associated with persistent, systemic, and tissue-specific T-cell dysregulation. These differences are important when considering how transcriptional regulators such as KLF family members may influence cardiovascular immunity.

### 4.1. Atherosclerosis: Chronic Imbalance Between Effector and Regulatory T Cells

Atherosclerosis represents the most extensively studied cardiovascular setting of adaptive immune activation. Within atherosclerotic plaques, Th1 cells, Th17 cells, CD8^+^ cytotoxic T cells, and Tregs coexist and exert divergent effects on lesion progression and stability [5,6]. Pro-inflammatory Th1 cells promote plaque inflammation largely through IFN-γ-mediated macrophage activation, whereas Th17 cells and CD8^+^ T cells may further contribute to inflammatory amplification, cytotoxic injury, and necrotic core expansion [5,40,41].

In contrast, Tregs exert protective effects by suppressing excessive immune activation, limiting inflammatory cytokine production, and promoting plaque stability [5,6,42]. The balance between plaque-infiltrating effector T cells and Tregs therefore represents a major determinant of inflammatory burden and lesion stability. Importantly, these effects are spatially organized within the plaque microenvironment and depend on crosstalk among T cells, macrophages, endothelial cells, and vascular smooth muscle cells.

### 4.2. Myocardial Infarction: Phase-Dependent Transition from Inflammation to Repair

T-cell responses after myocardial infarction are temporally dynamic and functionally heterogeneous. During the early inflammatory phase, activated CD4^+^ effector T cells and other inflammatory lymphocyte populations contribute to leukocyte recruitment and amplification of myocardial inflammation [43]. Excessive or prolonged effector activity during this phase may aggravate tissue injury and adverse remodeling.

As the immune response transitions toward resolution, Tregs accumulate in the infarcted myocardium and contribute to tissue repair by restraining excessive inflammation and supporting reparative macrophage phenotypes [7,8]. Cardiac-draining lymph nodes also participate in the generation of antigen-specific Tregs after infarction, indicating that post-MI immune regulation is coordinated across lymphoid and cardiac compartments rather than being confined to the injured myocardium [44]. Thus, the consequences of T-cell activity after MI depend not only on subset identity but also on the timing and anatomical location of the response.

### 4.3. Myocarditis: Antigen-Driven Pathogenic T-Cell Activation

Myocarditis represents a prototypical T-cell-mediated cardiac inflammatory disease. In experimental autoimmune myocarditis, autoreactive CD4^+^ T cells recognizing cardiac antigens are central drivers of myocardial inflammation and injury [9,45]. Th1- and Th17-skewed responses promote inflammatory cytokine production, recruitment of additional immune cells, and cardiomyocyte damage.

Protective mechanisms are largely mediated by regulatory pathways that restrain the activation and persistence of pathogenic effector T cells. Tregs contribute to immune suppression, whereas inhibitory checkpoint pathways help limit myocardial injury [46]. In particular, PD-1 signaling suppresses pathogenic T-cell responses and protects against immune-mediated cardiomyocyte damage [10]. Compared with other cardiovascular conditions, the link between antigen recognition, effector T-cell activation, and target-organ injury is especially direct in myocarditis.

### 4.4. Hypertension: Vascular and Renal T-Cell-Mediated Inflammation

Hypertension is increasingly recognized as an immune-mediated disorder in which activated T cell subsets contribute to vascular and end-organ injury [47]. CD4^+^ effector T cells, Th17 cells, and CD8^+^ T cells have been implicated in vascular inflammation, endothelial dysfunction, renal injury, and blood-pressure elevation, partly through cytokine-mediated activation of vascular and myeloid cells [47,48,49,50].

These immune responses are distributed across multiple anatomical compartments, including the vasculature, kidney, and secondary lymphoid tissues. Tregs may counteract hypertensive inflammation by restraining effector T cell activation and limiting vascular immune injury [51,52]. Chronic low-grade activation of these pathogenic T cell subsets sustains hypertensive pathology, emphasizing the importance of T cell subset balance in this condition [11,50].

### 4.5. Heart Failure: Stage-Dependent T-Cell Dysfunction and Adverse Remodeling

T-cell dysregulation in heart failure is heterogeneous and depends on disease etiology, stage, and inflammatory context. During earlier or reparative phases of cardiac injury, Tregs may suppress excessive inflammation, support tissue repair, and limit adverse remodeling [13]. However, under chronic ischemic or inflammatory conditions, Tregs may lose suppressive competence or acquire pro-inflammatory features.

At the same time, activated CD4^+^ effector T cells, Th17-like cells, and cytotoxic T-cell populations may contribute to persistent myocardial inflammation, fibroblast activation, fibrosis, and ventricular remodeling [12,53]. Thus, the functional consequences of T-cell responses in heart failure cannot be defined solely by subset identity; they must also be interpreted according to disease stage, tissue localization, and functional state.

**Figure 2:**
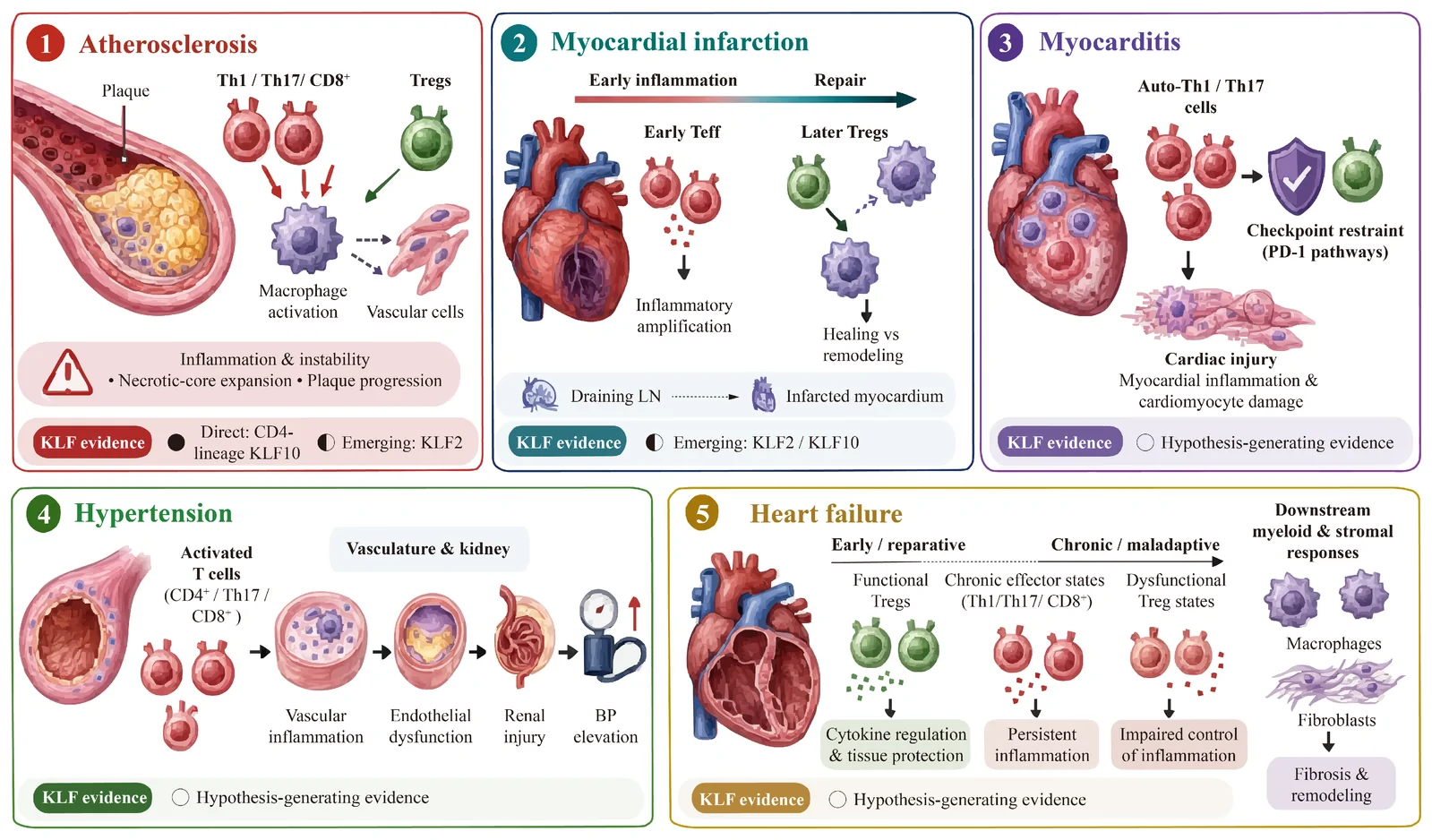
Disease-specific T-cell landscapes and KLF evidence across major cardiovascular diseases. T-cell functions vary according to subset identity, anatomical localization, disease stage, and interactions with cardiovascular, myeloid, and stromal cells. Across atherosclerosis, myocardial infarction, myocarditis, hypertension, and heart failure, effector T-cell responses contribute to inflammation and tissue injury, whereas regulatory T-cell programs may restrain inflammation or support repair in a context- and stage-dependent manner. The strength of evidence for KLF-dependent T-cell mechanisms differs substantially among diseases: direct disease-specific evidence is strongest for KLF10 manipulation within the CD4^+^ T-cell lineage in experimental atherosclerosis, with complementary functional evidence implicating Treg–macrophage interactions without establishing exclusively Treg-intrinsic causality; KLF2 in atherosclerosis and KLF2/KLF10 in myocardial infarction are supported by emerging mechanistic evidence; and proposed KLF-dependent mechanisms in myocarditis, hypertension, and heart failure remain hypothesis-generating. Filled, half-filled, and open circles denote direct disease-specific, emerging mechanistic, and hypothesis-generating evidence, respectively. Evidence grades refer specifically to KLF-dependent T-cell mechanisms rather than to the overall evidence for T-cell involvement in each disease. Arrows indicate the direction of the illustrated biological processes; panel colors distinguish disease contexts and do not denote evidence strength.

Together, these disease-specific differences provide the context for evaluating KLF-dependent T-cell programs according to disease stage, anatomical compartment, and strength of causal evidence. Figure 2 and Table 2 summarize these disease-specific T-cell landscapes and evidence levels.

## 5. Evidence Linking KLF-Dependent T-Cell Programs to Cardiovascular Disease

Consistent with the evidence framework introduced above, we classify cardiovascular evidence as direct disease-specific, emerging mechanistic, or hypothesis-generating according to the degree of disease- and T-cell-lineage-specific causal validation.

### 5.1. Atherosclerosis: Direct Evidence for KLF10 Within the CD4^+^ T-Cell Lineage

Among the cardiovascular diseases considered in this Review, atherosclerosis currently provides the strongest direct evidence linking a KLF-dependent T-cell program to disease pathology. Klf10 deletion within the CD4^+^ T-cell lineage exacerbates atherosclerotic lesion development, increases inflammatory cell infiltration, enlarges necrotic core formation, and impairs macrophage efferocytosis [54]. Complementary functional and adoptive-transfer experiments implicate Tregs and Treg–macrophage regulatory crosstalk in this phenotype [54]. However, because the genetic deletion was not restricted specifically to Tregs, these findings do not establish that the vascular phenotype results exclusively from Treg-intrinsic KLF10 deficiency.

The available evidence therefore supports a causal role for KLF10 within the CD4^+^ T-cell lineage in experimental atherosclerosis, with Treg-mediated mechanisms representing an important component of the observed phenotype. The relative contributions of Tregs versus other CD4^+^ T-cell populations remain to be defined. Further studies using Treg-restricted genetic models will be required to determine the extent to which altered suppressive activity, TGF-β-associated regulation, metabolic adaptation, and macrophage-directed signals account for the vascular phenotype.

KLF2 provides a strong mechanistic rationale for regulating T-cell participation in atherosclerosis because it controls lymphocyte quiescence, egress, recirculation, and chemokine-receptor expression [16,32,33]. These functions could influence the number and phenotype of T cells entering atherosclerotic lesions. However, direct evidence showing that T-cell-restricted manipulation of KLF2 alters atherosclerotic disease remains limited. Its role in atherosclerosis should therefore be classified as emerging mechanistic evidence rather than established disease causality.

Potential contributions of KLF4 and KLF13 are even less directly defined. KLF4-dependent regulation of IL-17-producing T cells and KLF13-dependent CCL5/RANTES induction may be relevant to chronic plaque inflammation [20,39]. Nevertheless, cardiovascular disease-specific studies demonstrating causal effects of these T-cell-intrinsic pathways are currently lacking, and their proposed roles in atherosclerosis remain hypothesis-generating.

### 5.2. Myocardial Infarction: Mechanistically Plausible but Incompletely Validated

T-cell responses after myocardial infarction are highly dependent on timing, tissue localization, and cellular phenotype. Tregs contribute to the resolution of inflammation, reparative macrophage responses, and myocardial healing, whereas excessive or prolonged effector T-cell activation may aggravate injury and adverse remodeling [14,55].

These observations provide a plausible context in which KLF2- and KLF10-dependent programs could influence post-infarction immunity. KLF2 may regulate the egress, recirculation, and myocardial recruitment of T-cell populations [16,32,33], whereas KLF10 may contribute to Treg suppressive competence and metabolic adaptation within the inflammatory infarct environment [17,19]. However, these proposed relationships have not been directly demonstrated using MI-specific T-cell-restricted KLF models. They should therefore be presented as emerging mechanistic hypotheses rather than established components of post-MI repair.

The potential involvement of KLF4- and KLF13-dependent inflammatory programs is even less certain. Although type-17/IL-17-associated inflammation has been implicated in post-infarction immune injury and adverse ventricular remodeling [56,57], providing a biologically plausible context for KLF4-dependent regulation of IL-17-producing T cells [20,38], direct evidence linking T-cell-intrinsic KLF4 or KLF13 activity to infarct size, immune resolution, cardiac repair, or ventricular remodeling is currently unavailable. These relationships should therefore be regarded as hypothesis-generating.

### 5.3. Myocarditis: Predominantly Hypothesis-Generating Relationships

Myocarditis is characterized by antigen-driven activation of pathogenic T cells, making transcriptional regulation of T-cell activation, differentiation, and immune restraint highly relevant to disease development [46]. On this basis, KLF10-mediated regulation of TGF-β responsiveness and Treg function could potentially limit pathogenic immunity, whereas loss of KLF2-dependent quiescence could facilitate inappropriate or sustained T-cell activation.

KLF4-dependent Th17 differentiation [20] and KLF13-dependent CCL5/RANTES production [39] may also be mechanistically relevant because Th17-skewed responses and inflammatory cell recruitment contribute to myocardial injury [9,45,46]. Nevertheless, direct studies demonstrating that T-cell-specific manipulation of KLF2, KLF4, KLF10, or KLF13 modifies myocarditis severity are lacking. Accordingly, the proposed KLF–T-cell relationships in myocarditis remain largely hypothesis-generating.

This limitation is particularly important because findings from general T-cell biology cannot automatically be extrapolated to autoreactive cardiac T cells. Disease-specific models will be required to determine whether individual KLFs affect antigen recognition, clonal expansion, tissue recruitment, effector differentiation, regulatory restraint, or persistence within the myocardium.

### 5.4. Hypertension: Indirect Evidence from Trafficking and Effector–Regulatory Balance

T-cell activation contributes to vascular inflammation, renal injury, endothelial dysfunction, and blood-pressure elevation in experimental hypertension [11,47,48,49,50]. These observations provide a biological context in which KLF-dependent control of T-cell trafficking and differentiation may be relevant.

KLF2 could influence the distribution and tissue recruitment of activated T cells through its regulation of lymphocyte homing and chemokine-receptor expression [16,32,33]. KLF10 may affect regulatory restraint through its effects on Treg function and metabolic fitness [17,19], whereas KLF4-dependent regulation of IL-17-producing T cells may be relevant to Th17-associated vascular inflammation [20].

However, these relationships are currently inferred from general T-cell mechanisms rather than demonstrated in T-cell-specific KLF models of hypertension. No individual KLF should therefore be described as an established regulator of hypertensive pathology at present. The available evidence supports mechanistic plausibility but not disease-specific causality.

### 5.5. Heart Failure: Context- and Stage-Dependent Inference

The potential relevance of KLF-dependent T-cell programs to heart failure is complicated by the heterogeneity of the syndrome. T-cell phenotypes vary according to disease etiology, stage, tissue compartment, and the transition from acute injury to chronic remodeling. Tregs may exert protective effects during reparative phases but can become quantitatively or functionally altered during chronic heart failure, whereas effector and cytotoxic T-cell populations may contribute to persistent inflammation, fibrosis, and ventricular remodeling [12,13,53].

KLF2 may theoretically influence the recruitment and anatomical distribution of these T-cell populations [16,32,33], whereas KLF10 may affect Treg suppressive function and metabolic adaptation [17,19]. KLF4- or KLF13-dependent inflammatory programs could also contribute under selected conditions [20,21,39]. However, these possibilities remain extrapolations from established T-cell biology and have not been causally validated in heart-failure-specific T-cell-restricted KLF models.

Moreover, a pathway that is protective during early tissue repair may have different consequences during chronic inflammation or fibrosis. Future studies should therefore evaluate KLF functions according to heart-failure etiology and disease stage rather than assuming a uniform effect across the syndrome.

### 5.6. Human Evidence and Translational Gaps

Human evidence directly linking KLF activity in defined T-cell subsets to cardiovascular disease phenotypes remains limited. Existing human studies have primarily characterized changes in T-cell abundance, phenotype, clonality, and tissue localization rather than demonstrating causal involvement of a specific KLF-dependent transcriptional program.

Single-cell and multimodal profiling of human carotid plaques has revealed marked heterogeneity among plaque-associated T cells, including activated, differentiated, exhausted, and plaque-enriched CD4^+^ and CD8^+^ T-cell states [58,59]. Paired plaque–blood T-cell receptor sequencing has further demonstrated plaque-specific clonal expansion of effector CD4^+^ T cells, supporting a potential antigen-driven component of human atherosclerosis [60]. Spatial transcriptomic studies have identified region-specific transcriptional programs and spatially organized immune niches within human atherosclerotic plaques [61,62]. However, these studies have not yet established a disease-relevant KLF-dependent T-cell program.

In human myocardium, single-cell, chromatin-accessibility, spatial, and T-cell receptor analyses have revealed distinct immune and tissue states associated with myocardial infarction, ischemic cardiomyopathy, and dilated cardiomyopathy [63,64]. Importantly, these human datasets describe T-cell states and potential regulatory programs but do not by themselves establish functional KLF activity or causality.

To date, no KLF-dependent T-cell signature has undergone prospective validation as a cardiovascular biomarker, prognostic indicator, or predictor of therapeutic response.

## 6. Integrated Molecular Mechanisms of the KLF–T-Cell Axis

KLF-dependent regulation of T-cell function is embedded within broader signaling networks that integrate antigen recognition, co-stimulation, cytokine exposure, nutrient availability, and tissue-derived stress signals. Rather than functioning as isolated lineage determinants, individual KLFs interact with pathways controlling quiescence, migration, differentiation, apoptosis, and metabolic adaptation. Importantly, the strength of evidence differs across these interactions. Some relationships, such as KLF2-dependent transcription of trafficking receptors and KLF13-dependent regulation of CCL5 and BCL-XL, are supported by direct molecular or genetic evidence [16,21,32,39], whereas other proposed connections reflect broader signaling contexts that have not yet been causally demonstrated in cardiovascular T cells.

### 6.1. Quiescence and Trafficking: The PI3K–Akt–FOXO1–KLF2 Axis

The balance between T-cell quiescence and activation is closely linked to growth-factor and nutrient-sensing pathways. In resting or naïve T cells, nuclear FOXO1 promotes the expression of KLF2 together with genes involved in lymphocyte homing and homeostasis. By contrast, T-cell receptor and co-stimulatory signaling activate PI3K–Akt and mTOR pathways, leading to FOXO1 phosphorylation and nuclear exclusion, reduced KLF2 expression, and remodeling of the trafficking-receptor repertoire. FOXO1 deletion reduces Klf2, CD62L, and CCR7 expression and disrupts naïve T-cell localization, supporting an upstream FOXO1–KLF2 transcriptional relationship [65,66].

KLF2 directly activates the promoters of CD62L and S1PR1, thereby coordinating lymph-node homing, lymphoid-organ egress, and peripheral recirculation [16,32]. PI3K–mTOR signaling can suppress this KLF2-dependent trafficking program after immune activation, linking metabolic activation to changes in immune-cell localization [66]. These mechanisms provide a plausible basis through which KLF2 may influence the entry and distribution of T cells in cardiovascular tissues. However, the experimental evidence supporting these trafficking mechanisms derives predominantly from non-cardiovascular T-cell models, and direct validation of their role in T-cell recruitment in specific cardiovascular diseases remains limited [16,66].

KLF2 has also been associated with maintenance of the quiescent transcriptional state through suppression of c-Myc-dependent activation programs [31]. Nevertheless, its physiological contribution to cell-cycle control appears to be context-dependent. Conditional deletion studies in postactivated CD8^+^ T cells found that endogenous KLF2 was required for appropriate S1PR1 and CD62L expression and trafficking but had little effect on proliferative behavior [34]. Thus, the trafficking function of KLF2 is more consistently established than a universal requirement for quiescence across all T-cell states [34].

### 6.2. TGF-β/SMAD–KLF10 Signaling and Immune Tolerance

TGF-β signaling is initiated through engagement of the type II and type I TGF-β receptors, followed by phosphorylation of SMAD2/3 and formation of transcriptional complexes with SMAD4 [67,68]. KLF10, originally identified as a TGF-β-inducible transcription factor, participates in this pathway at several levels. However, its molecular functions differ according to T-cell subset and should not be described as a single uniform Treg-specific mechanism.

In CD8^+^ T cells, KLF10 binds to the TGF-β receptor II promoter and promotes receptor expression, thereby enhancing cellular responsiveness to TGF-β after T-cell receptor stimulation [18]. In the CD4^+^ T-cell compartment, KLF10 has been linked to regulation of TGF-β1- and Foxp3-associated transcriptional programs, inducible Treg differentiation, and suppressive function [17]. These findings indicate that KLF10 participates in a reciprocal regulatory relationship with TGF-β signaling: TGF-β can induce KLF10, whereas KLF10 can reinforce selected components of TGF-β-responsive transcription. The direct downstream targets and functional consequences nevertheless vary among CD4^+^ conventional T cells, Tregs, and CD8^+^ T cells [69,70].

KLF10-dependent immune regulation also extends beyond canonical TGF-β/SMAD signaling. Klf10 deletion within the CD4^+^ T-cell lineage has been associated with impaired Treg mobilization, reduced mitochondrial respiration and glycolysis, and altered PI3K–Akt–mTOR activity under metabolic stress [19]. KLF10 may therefore connect cytokine-dependent regulatory programs with the metabolic and migratory competence required for Tregs to function in inflamed tissues [19].

### 6.3. Inflammatory Differentiation and Chemokine Output: KLF4 and KLF13

Effector T-cell differentiation is shaped by cytokine- and antigen-receptor-dependent signaling networks involving STAT proteins, NF-κB, AP-1, and lineage-associated transcription factors [71,72,73]. Within this broader context, KLF4 and KLF13 regulate distinct components of inflammatory T-cell responses.

KLF4 contributes to Th17-associated differentiation by directly binding to the *Il17a* promoter and promoting IL-17 expression [20,38]. T-cell-specific *Klf4* deletion reduces the generation of IL-17-producing CD4^+^ T cells and attenuates inflammatory disease in an experimental autoimmune model [38]. KLF4 therefore provides an additional transcriptional input into the Th17 program alongside the canonical STAT3–RORγt axis [73,74]. However, direct molecular interactions between KLF4 and STAT3, mTOR, or HIF-1α in cardiovascular T cells have not been firmly established and should be regarded as potential signaling convergence rather than a demonstrated linear pathway.

KLF13 regulates a distinct aspect of inflammatory T-cell activity by promoting the delayed transcriptional induction of CCL5/RANTES after T-cell activation [39,75]. This timing is consistent with a role in sustained or late-phase chemokine output rather than immediate early activation. Although CCL5 may contribute to leukocyte recruitment and persistent tissue inflammation, direct evidence linking T-cell-intrinsic KLF13 activity to cardiovascular inflammatory outcomes remains lacking [39]. Promoter-level cooperation between KLF13 and NF-κB has been demonstrated for CCL5/RANTES transcription, but this finding does not establish a linear NF-κB–KLF13 signaling pathway [75].

### 6.4. KLF13-Dependent Survival Restraint

In addition to its role in chemokine regulation, KLF13 exerts a mechanistically distinct survival-restraining function. Genetic and promoter-level studies indicate that KLF13 represses the anti-apoptotic protein BCL-XL, whereas *Klf13* deficiency is associated with reduced apoptosis and prolonged thymocyte survival [21]. Importantly, this evidence derives predominantly from thymocyte and related lymphoid models and does not establish that the same mechanism operates in mature effector T cells or cardiovascular tissues. The cardiovascular relevance of KLF13-dependent survival restraint therefore remains to be determined.

### 6.5. KLF-Dependent Immunometabolic Regulation of T-Cell Fate

Metabolic remodeling is not merely a consequence of T-cell activation but contributes directly to lineage differentiation and functional fate. Activated effector T cells typically increase glycolytic and anabolic metabolism, whereas memory T cells exhibit enhanced mitochondrial respiratory capacity; many Treg states also rely substantially on mitochondrial metabolism, although their metabolic requirements vary according to activation and tissue context [76,77,78]. The mTOR–HIF-1α pathway is particularly relevant to the Th17–Treg balance: HIF-1α-dependent glycolysis promotes Th17 differentiation, whereas disruption of this program favors Foxp3^+^ Treg generation [79].

Among the KLFs considered in this Review, KLF10 currently has the clearest direct connection to T-cell metabolic fitness. KLF10-deficient Tregs exhibit reduced mitochondrial respiration and glycolysis, impaired PI3K–Akt–mTOR signaling, and defective chemotactic function [19]. These findings indicate that effective Treg activity cannot be reduced to dependence on a single metabolic pathway; rather, regulatory cells require sufficient metabolic flexibility to support suppressive function and migration under inflammatory or metabolic stress.

The commonly used model in which Tregs depend uniformly on fatty-acid oxidation should also be interpreted cautiously. Although pharmacological experiments have associated fatty-acid oxidation with Treg differentiation, genetic disruption of CPT1A-dependent long-chain fatty-acid oxidation did not substantially impair Treg formation, and several effects of etomoxir were independent of CPT1A [80]. Mitochondrial respiration and metabolic flexibility may therefore be more informative concepts than assigning Tregs an exclusively fatty-acid-dependent phenotype.

Lipid and cholesterol metabolism provide an additional cardiovascularly relevant dimension. Increasing plasma-membrane cholesterol can enhance T-cell-receptor clustering and CD8^+^ T-cell effector function, whereas dyslipidemia and hyperlipidemia can induce metabolic, migratory, and functional adaptations in Tregs [81,82,83]. Nevertheless, direct evidence that KLF2, KLF4, KLF10, or KLF13 controls cholesterol metabolism in cardiovascular T cells is currently lacking. Cholesterol-dependent effects should therefore be presented as features of the cardiovascular metabolic environment that may modify KLF-regulated T-cell states, rather than as established downstream pathways of individual KLFs.

Overall, these metabolic interactions remain mechanistically relevant but incompletely validated as disease-specific causal pathways in cardiovascular T cells.

**Figure 3:**
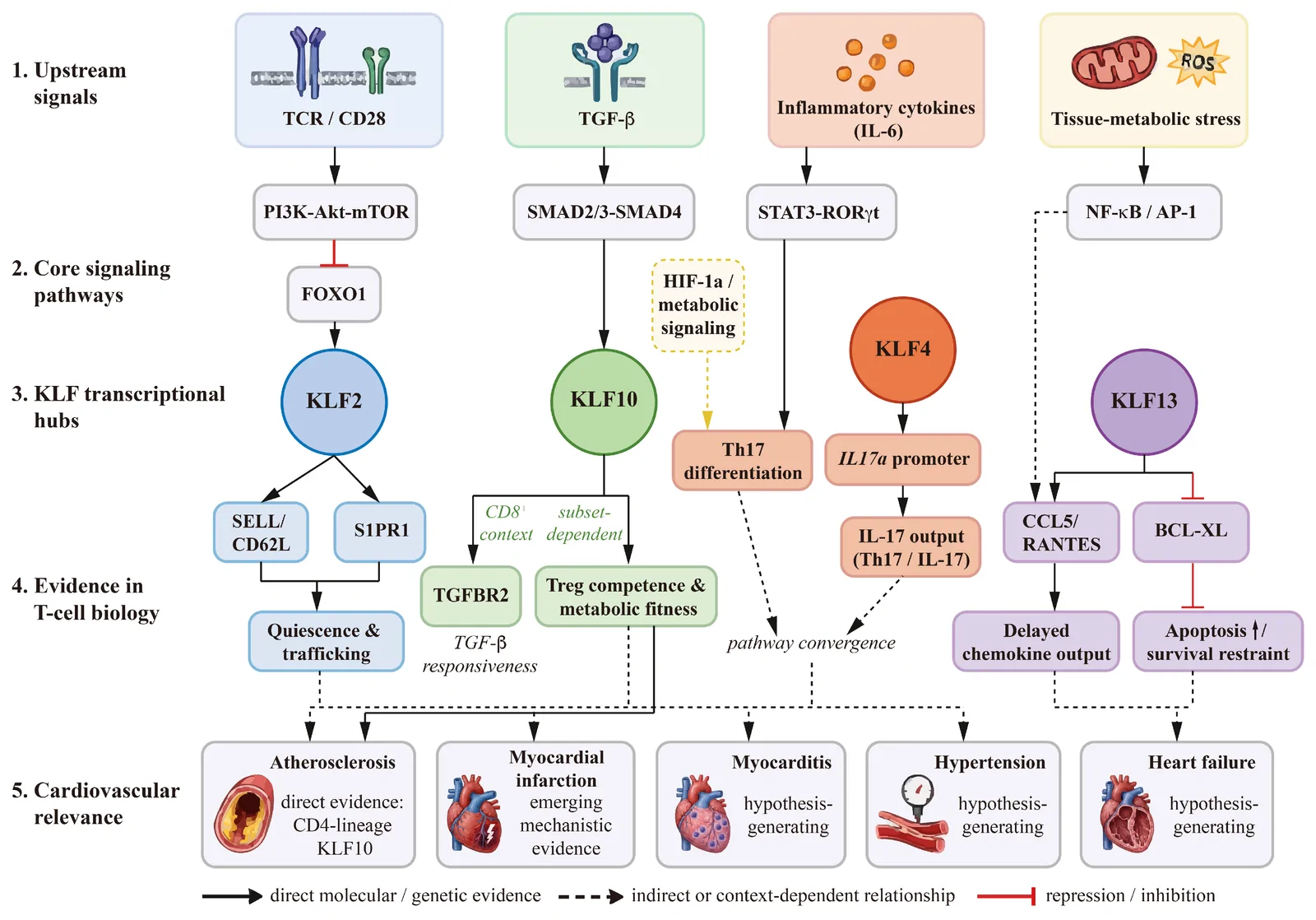
Core molecular signaling logic of the KLF–T-cell axis. Antigen and co-stimulatory signals, TGF-β, inflammatory cytokines, and tissue-metabolic stress converge on signaling pathways that shape distinct KLF-dependent T-cell programs. PI3K–Akt–mTOR signaling restrains FOXO1 and thereby influences the KLF2-dependent SELL/CD62L and S1PR1 trafficking program. TGF-β–SMAD2/3–SMAD4 signaling intersects with KLF10-dependent regulation of TGFBR2-mediated responsiveness in CD8^+^ T cells and with Treg suppressive competence and metabolic fitness in a subset-dependent manner. The canonical STAT3–RORγt pathway and KLF4-dependent *Il17a* transcription converge on Th17/IL-17 differentiation, while HIF-1α-associated metabolic signaling provides an additional context-dependent input into the Th17 program. KLF13 regulates delayed CCL5/RANTES expression and represses the anti-apoptotic protein BCL-XL, thereby promoting apoptosis-associated survival restraint; the latter evidence is derived predominantly from thymocyte models. At the cardiovascular disease level, direct disease-specific evidence is strongest for KLF10 manipulation within the CD4^+^ T-cell lineage in experimental atherosclerosis; evidence in myocardial infarction remains mechanistically emerging, whereas proposed KLF-dependent mechanisms in myocarditis, hypertension, and heart failure remain predominantly hypothesis-generating. This classification does not imply Treg-specific genetic causality. Solid arrows indicate directly supported molecular or genetic relationships, dashed arrows indicate indirect or context-dependent relationships or pathway convergence, and T-bars indicate repression or inhibition. Direct molecular relationships may derive from general T-cell models and should not be interpreted as cardiovascular disease-specific causal validation unless directly tested in the corresponding disease model. The upward arrow denotes increased apoptosis; colors distinguish the major KLF-centered modules and do not represent evidence strength.

The integrated molecular relationships of the KLF–T-cell axis are summarized in Figure 3.

## 7. Therapeutic Prospects and Translational Limitations

Therapeutic modulation of KLF-dependent T-cell programs may offer a means of altering several interconnected immune functions, including quiescence, trafficking, regulatory activity, inflammatory differentiation, and metabolic adaptation. However, direct targeting of KLF family members remains largely conceptual. Because KLFs are broadly expressed across immune and cardiovascular cell types, systemic manipulation may affect endothelial cells, macrophages, vascular smooth muscle cells, and other tissues in addition to T cells. Therefore, the therapeutic relevance of the KLF–T-cell axis should be evaluated separately from the broader feasibility of manipulating T-cell states.

### 7.1. Direct Modulation of KLF-Dependent T-Cell Programs

KLF2-oriented approaches could, in principle, reinforce quiescence-associated and homeostatic trafficking programs by modulating the expression of molecules such as CD62L and S1PR1. Such an approach might reduce inappropriate tissue recruitment of activated T cells. Nevertheless, increasing KLF2 activity systemically could also alter normal lymphocyte recirculation, immune surveillance, and responses to infection. Moreover, direct evidence that pharmacological enhancement of KLF2 improves cardiovascular outcomes through a T-cell-specific mechanism is currently lacking. KLF2 should therefore be regarded as a mechanistically supported but therapeutically unvalidated target [16,34,66].

KLF10-oriented strategies may be relevant to Treg suppressive competence, TGF-β responsiveness, and metabolic fitness [17,19]. Direct disease-specific evidence from experimental atherosclerosis derives from Klf10 manipulation within the CD4^+^ T-cell lineage, while complementary functional studies implicate Tregs and Treg–macrophage crosstalk without establishing exclusively Treg-intrinsic causality [54]. However, it remains uncertain whether increasing KLF10 expression or activity would reproduce the protective effects associated with intact endogenous KLF10 function. The downstream pathways responsible for the vascular phenotype have not been fully resolved. Although small-molecule inhibitors of KLF10 have been described experimentally [84], no KLF10-directed pharmacological strategy has been validated for cardiovascular immune therapy.

KLF4 and KLF13 currently represent less mature therapeutic candidates. Inhibiting KLF4-dependent inflammatory differentiation could theoretically reduce IL-17-associated immune responses [20,38], whereas modulation of KLF13 might influence CCL5/RANTES production [39]. However, both factors also participate in biological processes outside T cells, and the disease-specific consequences of their manipulation remain poorly defined. In particular, the pro-apoptotic or survival-restraining function of KLF13 raises the possibility that its inhibition could unintentionally prolong lymphocyte survival even if chemokine production were reduced [21].

### 7.2. Cell-Selective Delivery and Gene-Engineering Strategies

The principal challenge in targeting transcription factors is achieving cell-type and context specificity. Conventional systemic administration is unlikely to distinguish pathogenic T-cell subsets from protective immune populations or non-immune cardiovascular cells. Cell-selective delivery platforms, including antibody-directed nanoparticles, lipid-based carriers, and ligand-targeted systems, may enable the delivery of nucleic acids, small interfering RNAs, messenger RNAs, or gene-regulatory cargo to defined T-cell populations [85,86,87,88]. Preclinical studies have demonstrated targeted in vivo nucleic-acid delivery to T cells, including transient mRNA programming of antifibrotic CAR T cells in experimental cardiac injury [86,87,88]. However, these strategies remain technically demanding and require rigorous validation of targeting efficiency, intracellular delivery, tissue biodistribution, immunogenicity, and unintended effects on T-cell activation or phenotype [86,87,88].

Gene-editing and ex vivo engineering approaches may offer greater specificity. Primary human T cells can be modified by non-viral CRISPR–Cas9-mediated gene disruption or targeted knock-in, whereas CRISPR activation and interference systems permit programmable modulation of endogenous transcriptional programs [89,90]. Human Tregs have also been engineered with antigen-specific chimeric receptors, demonstrating the feasibility of enhancing tissue targeting and suppressive activity through ex vivo modification [91,92]. T cells or Tregs could therefore, in principle, be modified outside the body to enhance selected KLF-dependent regulatory programs before adoptive transfer. Nevertheless, permanent alteration of transcription-factor networks raises concerns regarding lineage instability, uncontrolled expansion, altered trafficking, unintended effector differentiation, and long-term off-target effects [91,92]. For cardiovascular indications, the anticipated benefit would need to justify the complexity, cost, and individualized manufacturing requirements of engineered cell products.

An alternative strategy may be to target downstream effectors rather than the KLF proteins themselves. For example, modulation of trafficking receptors, cytokine-responsive pathways, metabolic regulators, or chemokine outputs may be more pharmacologically tractable than direct manipulation of a zinc-finger transcription factor. Such downstream targeting could also permit greater temporal control, although it may not reproduce the coordinated effects of the upstream KLF program. Importantly, current studies establish the feasibility of T-cell-selective nucleic-acid delivery and ex vivo immune-cell engineering but do not directly validate therapeutic modulation of a KLF-dependent T-cell program in cardiovascular disease.

### 7.3. Treg-Directed Therapies as Adjacent Evidence of Therapeutic Tractability

Treg-directed interventions demonstrate that regulatory T-cell abundance and function can be therapeutically manipulated, but they should not be interpreted as direct evidence supporting KLF10-targeted therapy. Adoptive transfer of Tregs has improved cardiac function, reduced fibrosis, and promoted inflammatory resolution in experimental myocardial infarction models [14,93]. Similarly, low-dose IL-2 can preferentially expand and activate Tregs in selected clinical settings [94]. These findings support the broader therapeutic tractability of regulatory T-cell states.

However, neither Treg transfer nor low-dose IL-2 directly modulates KLF10, and neither establishes the efficacy or safety of KLF10-oriented intervention. Their relevance to the KLF–T-cell axis is therefore indirect. They provide proof that Treg number or function can be altered therapeutically, while leaving unresolved whether selective manipulation of KLF10 would offer additional efficacy, improved tissue targeting, or acceptable safety.

Engineered Treg strategies may provide a more direct bridge between these fields. Antigen-specific CAR-Tregs have shown enhanced targeting and suppressive activity in preclinical transplantation models [91,92]. Ex vivo modification could therefore potentially reinforce KLF10-associated suppressive or metabolic programs, enhance tissue homing or improve persistence after transfer. Nevertheless, the optimal molecular target, durability of the engineered phenotype, and risk of functional conversion remain uncertain [95]. These approaches should therefore be classified as preclinical or emerging rather than established KLF-based therapies.

### 7.4. Safety Considerations and Translational Barriers

Several barriers must be addressed before KLF-dependent T-cell programs can be considered clinically actionable. First, KLF family members are expressed and function in multiple immune and non-immune cell types. For example, KLF4 regulates monocyte differentiation, KLF13 participates in cardiac development, and T-cell KLF10 deficiency produces systemic metabolic consequences [19,96,97]. Broad systemic manipulation could therefore cause unintended vascular, hematopoietic, metabolic, or developmental effects.

Second, the same KLF may exert different effects according to T-cell subset, activation state, tissue environment, and disease stage. A pathway that supports acute inflammatory resolution may be neutral or detrimental during chronic immune suppression. Excessive reinforcement of quiescence or regulatory activity could impair antimicrobial or antitumor immunity, whereas inhibition of inflammatory programs could alter normal immune surveillance. These consequences should presently be regarded as mechanism-based safety concerns rather than clinically demonstrated effects of KLF-targeted therapy.

Conversely, inhibition of a survival-restraining factor such as KLF13 could unintentionally prolong lymphocyte survival because KLF13 represses the anti-apoptotic protein BCL-XL in thymocytes [21]. Most disease-specific evidence remains derived from animal models, whereas direct human validation of KLF activity in defined cardiovascular T-cell populations is limited.

Accordingly, future therapeutic development should prioritize reversible and cell-selective modulation, disease-stage-specific intervention, and rigorous evaluation of systemic immune consequences. At present, the most realistic translational path may involve identifying druggable downstream effectors or engineering T-cell products ex vivo rather than broadly activating or inhibiting individual KLF proteins.

**Figure 4:**
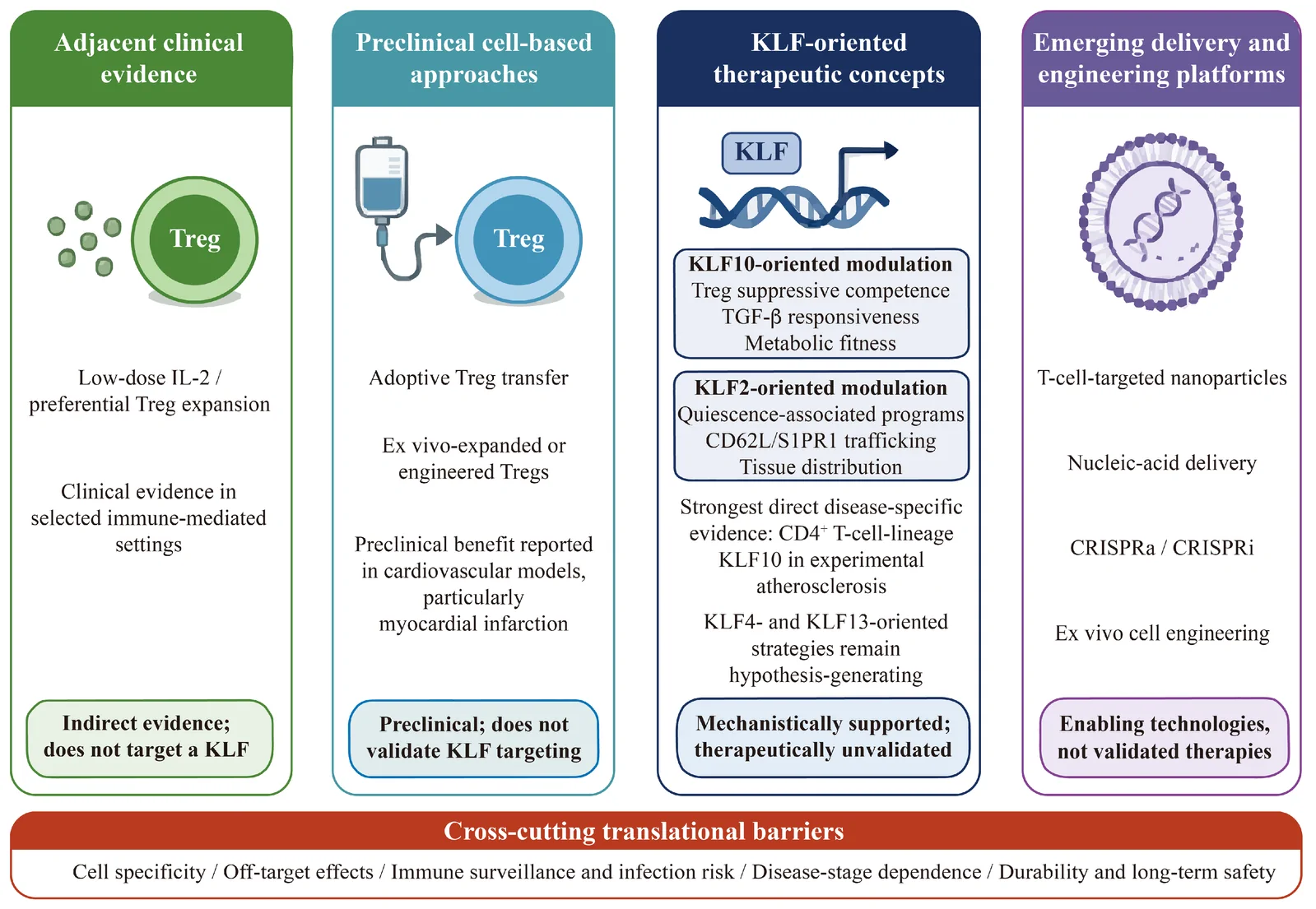
Translational framework for therapeutic strategies related to the KLF–T-cell axis. Therapeutic strategies are organized into complementary evidence and translational domains rather than a linear sequence of maturity. Low-dose IL-2 provides adjacent clinical evidence that preferential Treg expansion is therapeutically tractable in selected immune-mediated settings but does not directly target a KLF. Adoptive and engineered Treg approaches provide predominantly preclinical evidence that regulatory T-cell states can be therapeutically manipulated, including in cardiovascular injury models. KLF-oriented strategies remain therapeutically unvalidated: the strongest direct disease-specific evidence derives from KLF10 manipulation within the CD4^+^ T-cell lineage in experimental atherosclerosis, whereas KLF2 is supported primarily by emerging mechanistic evidence and KLF4- and KLF13-oriented concepts remain hypothesis-generating. T-cell-targeted nanoparticles, nucleic-acid delivery, CRISPR-based regulation, and ex vivo cell engineering represent enabling technologies rather than validated KLF-directed therapies. Cross-cutting translational barriers include cell- and subset-specific targeting, off-target effects, disruption of immune surveillance, infection risk, disease-stage dependence, durability, and long-term safety. Importantly, Treg-directed interventions demonstrate the therapeutic tractability of regulatory T-cell states but do not establish the efficacy or safety of direct KLF-targeted therapy.

The complementary evidence and translational domains are illustrated in Figure 4, while their target specificity, evidence basis, and principal barriers are compared in Table 3.

## 8. Challenges and Future Directions

Despite increasing interest in the KLF–T-cell axis, several limitations currently restrict its interpretation in cardiovascular disease. Much of the available evidence is derived from general lymphocyte studies conducted outside cardiovascular settings or from cardiovascular studies focused predominantly on endothelial cells, vascular smooth muscle cells, and myeloid populations [24,25,98]. Consequently, established functions of KLFs in T-cell quiescence, trafficking, differentiation, chemokine production, or regulatory activity cannot automatically be interpreted as causal mechanisms in individual cardiovascular diseases. Future studies should therefore prioritize disease-, stage-, tissue-, and T-cell-subset-specific validation.

### 8.1. Establishing Causality and Context Specificity

A major challenge is to distinguish T-cell-intrinsic KLF functions from developmental, systemic, and non-immune effects. Global or constitutive deletion of a KLF may produce major developmental or systemic phenotypes, including vascular or epithelial abnormalities, before cardiovascular disease is induced [99,100]. Even conventional T-cell-specific models may not fully separate developmental effects from functions in mature peripheral T cells. Conditional and inducible deletion strategies can help distinguish developmental functions from those operating after T-cell activation [34].

Inducible and lineage-restricted models will therefore be essential for determining whether individual KLFs act during T-cell development, activation, tissue recruitment, regulatory differentiation, or chronic persistence. These models should be combined, where appropriate, with adoptive-transfer, rescue, and gain- and loss-of-function experiments. Importantly, KLF activity should be evaluated separately across atherosclerosis, myocardial infarction, myocarditis, hypertension, and heart failure because the consequences of the same transcriptional program may vary according to disease stage, tissue compartment, and T-cell subset.

Context dependence is particularly relevant to KLF13. Its survival-restraining effect has been demonstrated predominantly in thymocytes and cannot be assumed to operate uniformly in mature effector T cells or cardiovascular tissues [21]. Similar caution is required when extrapolating KLF10-associated Treg mechanisms or KLF2-dependent trafficking programs across different diseases [16,17,18,19,32,33].

### 8.2. Spatial, Molecular, and Human Validation

Future analyses should integrate single-cell RNA sequencing, single-cell chromatin accessibility, spatial transcriptomics, T-cell receptor sequencing, and multiplex imaging with protein-level and functional validation. Regulon-inference approaches such as SCENIC may identify candidate KLF-associated T-cell states, but direct transcriptional regulation should be validated using approaches such as CUT&Tag, chromatin immunoprecipitation, promoter analysis, and targeted genetic perturbation [101,102], thereby distinguishing direct KLF-dependent regulation from broader signaling convergence.

Metabolic observations should likewise be linked to functional outcomes. Changes in glycolysis, mitochondrial respiration, or lipid metabolism are meaningful only when connected to T-cell suppression, inflammatory differentiation, migration, survival, or tissue persistence under cardiovascularly relevant conditions [76,103].

Future studies should examine KLF protein expression, localization, regulon activity, and downstream target signatures in defined T-cell subsets from both blood and diseased cardiovascular tissues. Longitudinal cohorts will be particularly important for determining whether altered KLF activity precedes disease progression, represents a secondary response to tissue injury, or predicts clinical outcome or treatment response. Species differences should also be considered when translating findings from murine thymocytes or experimental cardiovascular models to mature human T-cell populations [104].

### 8.3. Translational Prioritization and Safety

Given the limited disease-specific and therapeutic evidence, translational development should prioritize KLF-dependent pathways supported by direct disease-specific causality and interventions that are reversible, cell-selective, and disease-stage specific. Broad systemic manipulation of KLFs is unlikely to provide adequate specificity because individual family members also function in non-T-cell immune and cardiovascular populations [96,105,106]. Where direct transcription-factor targeting is impractical, validated downstream effectors may provide more pharmacologically tractable alternatives.

Future translational programs should establish predefined go/no-go criteria based on target engagement, T-cell and subset specificity, off-target immune effects, infection and antitumor surveillance, durability, and human relevance. Candidate strategies should progress from mechanistic plausibility to disease-specific causal validation, followed by evaluation in human cardiovascular T-cell populations before clinical translation.

The principal unresolved questions, methodological considerations, and research priorities are summarized in Table 4.

## 9. Conclusions

KLF family members regulate distinct and partially overlapping aspects of T-cell biology, including quiescence, trafficking, regulatory function, inflammatory differentiation, chemokine production, apoptosis, and metabolic adaptation. Among the family members discussed in this Review, KLF2 is most consistently associated with T-cell quiescence and immune trafficking, whereas KLF10 supports Treg suppressive function, TGF-β responsiveness, and metabolic competence. KLF4 contributes to IL-17-associated effector differentiation, while KLF13 regulates delayed CCL5/RANTES expression and, in thymocyte models, exerts a survival-restraining effect through apoptosis-related pathways.

However, the cardiovascular relevance of these T-cell-intrinsic programs is supported by markedly different levels of evidence. The strongest direct disease-specific evidence currently concerns KLF10 function within the CD4^+^ T-cell lineage in experimental atherosclerosis, with complementary functional studies implicating Tregs and Treg–macrophage interactions without establishing exclusively Treg-intrinsic causality. By contrast, the proposed involvement of KLF2, KLF4, KLF10, and KLF13 in myocardial infarction, myocarditis, hypertension, and heart failure is based largely on established T-cell biology and disease-associated immune mechanisms rather than direct causal validation in disease-specific T-cell-restricted models.

Future progress will require inducible and subset-specific genetic models, spatially and temporally resolved immune profiling, direct identification of KLF transcriptional targets, and validation in human cardiovascular tissues and longitudinal patient cohorts. Although KLF-dependent T-cell programs provide a useful framework for understanding cardiovascular immune regulation, direct therapeutic targeting remains preliminary and will require cell-selective delivery, careful assessment of disease stage, and rigorous evaluation of systemic immune and off-target effects.

## Figures and Tables

**Table 1:** Prioritized KLF family members and their principal functions in T-cell biology.

KLF Member	Principal T-Cell Context	Representative Molecular Targets and Functional Programs	Cardiovascular Evidence
KLF2	Resting/naïve and recirculating T cells	SELL/CD62L and S1PR1 expression; quiescence-associated transcription; lymph-node homing, egress, and peripheral recirculation	**Emerging mechanistic evidence.** Established regulation of T-cell quiescence and trafficking provides a plausible link to cardiovascular inflammation, but direct validation in disease-specific T-cell-restricted models remains limited.
KLF10	Tregs and TGF-β responsive CD4^+^ and CD8^+^ T cells	Treg suppressive competence; TGF-β-responsive transcription; TGFBR2 regulation in CD8^+^ T cells; metabolic and migratory fitness	**Direct disease-specific evidence.** Klf10 deletion within the CD4^+^ T-cell lineage causally alters experimental atherosclerosis. Treg-focused functional studies implicate Treg–macrophage interactions but do not establish exclusively Treg-intrinsic causality. Its proposed roles in myocardial infarction and heart failure remain mechanistically plausible but unvalidated.
KLF4	IL-17-producing CD4^+^ T cells	Direct regulation of the Il17a promoter; Th17-associated differentiation and inflammatory output	**Hypothesis-generating evidence.** KLF4-dependent IL-17 regulation may be relevant to cardiovascular inflammation, but direct cardiovascular disease-specific validation is lacking.
KLF13	Activated T cells in chemokine-regulation studies; thymocytes in survival studies	Delayed CCL5/RANTES induction; BCL-XL repression; apoptosis-associated survival restraint	**Hypothesis-generating evidence.** KLF13 may influence chemokine-driven inflammation, but direct cardiovascular validation is lacking. Survival-related findings are derived predominantly from thymocyte models.

**Note:** Evidence levels refer specifically to KLF-dependent T-cell mechanisms in cardiovascular disease and not to the general involvement of T cells in the indicated conditions. Direct disease-specific evidence denotes causal findings from cardiovascular models involving manipulation of a KLF within a defined T-cell lineage; emerging mechanistic evidence denotes established T-cell functions without sufficient disease-specific causal validation; hypothesis-generating evidence denotes relationships inferred primarily from general T-cell biology.

**Table 2:** Disease-specific T-cell programs across major cardiovascular diseases.

Disease Context	Predominant T-Cell Programs	Spatial and Temporal Context	Major Cellular Interactions	Principal Immunopathologic Consequences
Atherosclerosis	Th1, Th17, and CD8^+^ effector T cells; Tregs	Atherosclerotic plaque and adventitia; chronic immune activation	Macrophages, endothelial cells, and vascular smooth muscle cells	Effector T-cell programs amplify plaque inflammation, cytotoxic injury, and necrotic-core expansion, whereas Tregs restrain excessive inflammation and support plaque stability [5,6,40,41,42].
Myocardial infarction	Activated CD4^+^ effector T cells during early inflammation; regulatory and repair-associated Tregs during resolution	Cardiac-draining lymph nodes and infarcted myocardium; transition from inflammation to repair	Macrophages, fibroblasts, and stromal cells	Early or excessive effector responses may aggravate myocardial injury, whereas later Treg responses promote inflammatory resolution, reparative macrophage activity, and tissue healing [7,8,43,44].
Myocarditis	Autoreactive Th1- and Th17-skewed CD4^+^ T cells; Tregs and checkpoint-regulated T-cell states	Lymphoid activation followed by myocardial infiltration; antigen-driven response	Antigen-presenting cells, macrophages, and cardiomyocytes	Autoreactive effector T cells drive myocardial inflammation and cardiomyocyte injury, whereas Tregs and inhibitory checkpoint pathways restrain pathogenic activation [9,10,45,46].
Hypertension	CD4^+^ effector T cells, Th17 cells, and CD8^+^ T cells; Tregs	Vasculature and kidney; persistent low-grade immune activation	Vascular cells, renal cells, and myeloid cells	Effector T-cell activity contributes to endothelial dysfunction, vascular and renal inflammation, and blood-pressure elevation, whereas Tregs may provide regulatory restraint [11,47,48,49,50,51,52].
Heart failure	Effector CD4^+^, Th17-like, and cytotoxic T-cell populations; functional or dysfunctional Treg states	Failing myocardium; effects vary with etiology and disease stage	Macrophages, fibroblasts, and cardiomyocytes	Functional Tregs may support inflammatory resolution and repair during earlier phases, whereas chronic effector activation and Treg dysfunction contribute to persistent inflammation, fibrosis, and adverse remodeling [12,13,53].

**Note:** The functional effects of individual T-cell subsets are context-, tissue-, and stage-dependent and should not be interpreted as uniformly protective or pathogenic across cardiovascular diseases.

**Table 3:** Therapeutic strategies related to the KLF–T-cell axis: target specificity, evidence maturity, and translational barriers.

Strategy	Directly Targets a KLF?	Mechanistic Rationale	Evidence Maturity and Cardiovascular Basis	Major Translational Barriers
KLF10-oriented modulation	Yes (proposed)	Preserve or enhance KLF10-dependent Treg suppressive competence, TGF-β responsiveness, and metabolic or migratory fitness	**Mechanistically supported but therapeutically unvalidated.** Direct disease-specific evidence derives from KLF10 manipulation within the CD4^+^ T-cell lineage in experimental atherosclerosis; Treg-specific causality and therapeutic efficacy remain unestablished.	Lack of validated selective modulators; cell- and subset-specific delivery; incomplete definition of downstream effectors; potential effects in non-T-cell populations.
KLF2-oriented modulation	Yes (proposed)	Reinforce quiescence-associated programs and modulate SELL/CD62L- and S1PR1-dependent trafficking and tissue distribution	**Mechanistically supported but therapeutically unvalidated.** Cardiovascular relevance is inferred mainly from established T-cell trafficking biology rather than disease-specific therapeutic studies.	Risk of disrupting normal lymphocyte recirculation and immune surveillance; broad expression of KLF2; lack of selective delivery.
KLF4- or KLF13-oriented modulation	Yes (proposed)	Modulate IL-17-associated differentiation through KLF4 or delayed CCL5/RANTES output and survival restraint through KLF13	**Hypothesis-generating.** Direct cardiovascular disease-specific therapeutic evidence is lacking.	Limited disease-specific validation; context-dependent functions; potential unintended effects on inflammatory differentiation, apoptosis, and lymphocyte survival.
Adoptive or engineered Treg therapy	No	Increase regulatory-cell abundance or enhance suppressive, tissue-homing, and reparative properties before transfer	**Predominantly preclinical in cardiovascular disease.** Benefit has been reported in experimental cardiovascular injury models, particularly myocardial infarction.	Manufacturing complexity; lineage and functional stability; tissue homing; durability; cost and scalability.
Low-dose IL-2-mediated Treg expansion	No	Preferentially expand regulatory T-cell populations and strengthen regulatory immune states	**Clinical or translational evidence exists in selected immune-mediated settings; cardiovascular evidence remains limited.** This strategy demonstrates Treg tractability but does not validate KLF10 targeting.	Incomplete Treg specificity; dose optimization; variable durability; expansion of non-Treg IL-2-responsive populations.
T-cell-selective delivery and gene-engineering platforms	Potentially	Deliver nucleic acids, CRISPRa/CRISPRi cargo, or other regulatory molecules to defined T-cell subsets or engineer cells ex vivo	**Emerging enabling technologies, not validated KLF-directed cardiovascular therapies.**	Delivery efficiency; off-target editing or expression; immunogenicity; manufacturing; long-term safety and regulatory complexity.
Downstream effector targeting	No	Modulate more tractable downstream pathways, such as trafficking receptors, cytokine-responsive programs, metabolic regulators, or chemokine outputs	**Evidence varies according to the selected target.** May offer greater pharmacological feasibility than direct transcription-factor targeting.	Partial reproduction of the upstream KLF program; pathway redundancy; disease- and stage-dependent effects.

**Note:** Treg-directed interventions are included as adjacent evidence that regulatory T-cell states are therapeutically tractable; they do not directly target a KLF and should not be interpreted as validation of KLF-directed therapy. Evidence levels refer specifically to cardiovascular applications of KLF-dependent T-cell modulation.

**Table 4:** Priority research questions for advancing the KLF–T-cell field in cardiovascular disease.

Priority Area	Core Unresolved Questions	Recommended Approaches	Key Interpretive Considerations	Anticipated Contribution
Disease- and T-cell-subset-specific causality	Does inducible, T-cell-intrinsic manipulation of individual KLFs alter the development or progression of specific cardiovascular diseases? At which disease stage and in which T-cell subset are these effects most relevant?	Inducible and lineage-restricted genetic models; adoptive transfer; mixed bone-marrow chimeras; gain- and loss-of-function studies; rescue experiments; longitudinal cardiovascular phenotyping	Developmental, systemic, and non-T-cell effects must be distinguished from functions in mature peripheral T cells	Establish causal, subset-specific, and stage-dependent functions of individual KLFs
Spatial and temporal immune mapping	Where and when are KLF-associated T-cell states present across blood, lymphoid tissues, plaques, myocardium, vasculature, and kidney? How do these states evolve during inflammation, repair, and chronic remodeling?	Reanalysis of existing cardiovascular immune atlases; scRNA-seq; scATAC-seq; paired scTCR-seq; spatial transcriptomics; multiplex imaging	KLF transcript abundance alone does not establish transcription-factor activity or functional relevance	Define the anatomical localization, clonal structure, and temporal dynamics of KLF-associated T-cell programs
Direct transcriptional targets and pathway integration	Which genes are directly regulated by KLF2, KLF4, KLF10, and KLF13 in defined T-cell subsets? How do these programs intersect with TGF-β/SMAD, PI3K–Akt–mTOR, STAT3, NF-κB/AP-1, and HIF-1α-associated signaling?	CUT&Tag or ChIP-seq; ATAC-seq; promoter and reporter assays; transcriptomic profiling; targeted genetic perturbation; downstream rescue experiments	Direct promoter occupancy and causal target function should be distinguished from indirect pathway convergence	Define causal transcriptional networks and identify potentially tractable downstream effectors
Immunometabolism and intercellular crosstalk	How do KLF-dependent programs—particularly KLF10-associated regulation—affect glycolysis, mitochondrial respiration, and lipid metabolism? How do altered T-cell states influence macrophages, endothelial cells, fibroblasts, and other stromal populations?	Extracellular flux analysis; metabolomics; stable-isotope tracing; lipidomics; co-culture and organotypic systems; ligand–receptor analysis; spatial multi-omics	Metabolic changes should be linked to functional outcomes rather than interpreted as inherently protective or pathogenic	Identify metabolic and intercellular mediators of inflammation, immune resolution, and tissue remodeling
Human validation and clinical relevance	Are KLF-associated regulons and downstream signatures detectable in defined human T-cell subsets? Are they associated with disease stage, clinical outcome, or treatment response?	Analysis of blood and cardiovascular tissues; protein and subcellular-localization assays; regulon analysis; longitudinal patient cohorts; validation across independent datasets	Species differences and cross-sectional associations limit causal interpretation; mRNA expression alone is insufficient	Establish human relevance and evaluate potential biomarkers or patient-stratification signatures
Therapeutic feasibility, delivery, and safety	Can KLF-dependent T-cell programs be modulated selectively, reversibly, and safely? Is targeting a downstream effector more feasible than manipulating the transcription factor itself?	T-cell-selective delivery; nucleic-acid-based modulation; CRISPRa/CRISPRi; ex vivo cell engineering; downstream-effector targeting; preclinical efficacy and safety studies	Cell specificity, off-target effects, immune surveillance, infection risk, disease-stage dependence, durability, and long-term safety must be evaluated	Define feasibility and go/no-go criteria for future KLF-oriented cardiovascular interventions

## Data Availability

No new data were created or analyzed in this study. Data sharing is not applicable to this article.

## Abbreviations

The following abbreviations are used in this manuscript:

CCL5	C-C motif chemokine ligand 5
CVD	Cardiovascular disease
Foxp3	Forkhead box P3
IFN-γ	Interferon-γ
IL	Interleukin
KLF	Krüppel-like factor
MI	Myocardial infarction
PD-1	Programmed cell death protein 1
S1PR1	Sphingosine-1-phosphate receptor 1
Teff	Effector T cell
TGF-β	Transforming growth factor-β
Th1	T helper 1 cell
Th17	T helper 17 cell
Treg	Regulatory T cell
